# Differential roles of highly expressed PFKFB4 in colon adenocarcinoma patients

**DOI:** 10.1038/s41598-023-43619-4

**Published:** 2023-09-28

**Authors:** Xiaojing Gu, Xingchen Dai, Yongli Huang, Yuhuan Zhang, Lintao Dong, Chanchan Gao, Fang Wang

**Affiliations:** 1 Department of Gastroenterology, General Hospital, Ningxia Medical University, Yinchuan, Ningxia China; 2 School of Clinical Medicine, Ningxia Medical University, Yinchuan, Ningxia China; 3 Key Laboratory of Fertility Preservation and Maintenance of Ministry of Education, Department of Biochemistry and Molecular Biology, School of Basic Medical Sciences, Ningxia Medical University, Yinchuan, Ningxia China; 4 Department of Oncology, Zhongda Hospital, Southeast University, Nanjing, Jiangsu China

**Keywords:** Colorectal cancer, Cancer metabolism, Oncogenes

## Abstract

Colon adenocarcinoma (COAD) is a common malignant tumor, and the role of the protein PFKFB4 in glycolysis and pentose phosphate pathways is crucial. Researchers investigated the clinical significance of PFKFB4 in COAD by studying its expression in 79 tissue samples using immunohistochemistry. We found that PFKFB4 expression was significantly higher in COAD patients, particularly in the sigmoid colon. Interestingly, high PFKFB4 expression was associated with both improved overall survival (OS) and post-progression survival (PPS) in COAD patients. Further analysis revealed that genes associated with PFKFB4 were linked to various metabolic pathways, including amino acid biosynthesis, glycolysis, gluconeogenesis, glucose metabolism, and inflammatory response. PFKFB4 expression also showed correlations with the infiltration of different immune cell types in COAD patients, such as CD8+ T cells, CD4+ T cells, regulatory T cells (Tregs), macrophages, neutrophils, dendritic cells, active mast cells, and resting NK cells. Overall, the relationship between PFKFB4 expression and the prognosis of COAD is complex and diverse, possibly playing different roles at different stages of the disease. Moreover, its mechanism might involve interactions with various metabolic pathways and immune infiltration in the tumor microenvironment. These findings provide valuable insights into the potential role of PFKFB4 as a biomarker or therapeutic target in COAD.

## Introduction

Glycolysis is a crucial metabolic process that takes place in human cells when glucose is broken down. It produces substrates essential for various metabolic pathways, including fatty acid and cholesterol synthesis, the pentose phosphate pathway (PPP), and the tricarboxylic acid cycle (TCA)^1^. The Warburg effect, also known as aerobic glycolysis, is a phenomenon in which tumor cells, even in the presence of oxygen, have a preference for metabolizing glucose through glycolysis instead of mitochondrial oxidative phosphorylation (OXPHOS), leading to significant lactic acid production^2,3^.

Phosphofructokinase 2 (PFK2) is a bifunctional enzyme with both kinase and phosphatase activities. In humans, four PFK2 isozymes exist: PFKFB1, PFKFB2, PFKFB3, and PFKFB4^4^. Among these isozymes, phosphoric acid fructose kinase 1, phosphofructo-2-kinase/fructose-2,6-biphosphatase 4 (PFKFB4) plays a vital role in regulating the flux through the glycolytic and pentose phosphate pathways and ATP synthesis. It accomplishes this by controlling the levels of fructose 2,6-bisphosphate, the most potent allosteric effector. These pathways and ATP synthesis significantly impact tumor development^5,6^.

Studies have demonstrated that PFKFB4 is highly expressed in various types of human cancers, such as bladder cancer^7^, prostatic small cell neuroendocrine carcinoma (SCNC)^8^, pancreatic cancer, gastric cancer^9^, glioblastoma^10^, and acute myeloid leukemia (AML)^11^. Colon adenocarcinoma (COAD) is a prevalent form of cancer worldwide. Recent epidemiological data ranks it as the fourth most common cancer in China, with the fifth and fourth highest mortality rates for males and females, respectively^12^. However, the role of PFKFB4 in COAD remains unclear. Therefore, this study aimed to investigate the association between PFKFB4 expression levels and the clinicopathological characteristics and prognosis of COAD patients. Additionally, we analyzed co-expression gene signatures and immune infiltration features related to PFKFB4 expression using public databases to explore the potential function of PFKFB4 in COAD.

## Results

### Clinicopathological characteristics of patients with COAD

A total of 79 COAD patients were included in the study, comprising 43 women and 36 men, with a median age of 62 years (range, 27–81 years). Among them, 45 cases involved the colon as the tumor site, while 34 cases were associated with the sigmoid colon. The mean tumor diameter was 4.50 ± 0.2 cm, ranging from 1 to 10 cm. Serosa involvement was absent in 13 cases, and 27 cases exhibited lymph node metastases. Tumor differentiation was categorized as well, moderate, or poor in 1, 40, and 27 cases, respectively. Distant metastases were observed in 3 out of the 79 cases. Dukes' stage classification included 11 cases at stage A, 38 cases at stage B, 26 cases at stage C, and 3 cases at stage D. Detailed clinicopathological profiles of the patients are summarized in Table 1. Based on the above data, the relationship between PFKFB4 expression and various clinicopathological features can be obtained. PFKFB4 expression significantly differed between colon and sigmoid colon cases (*p* = 0.0210). However, age, gender, tumor size, differentiation, pT stage, pN stage, and pM stage did not show significant associations with PFKFB4 expression. Dukes’ stage had a trend toward an association with PFKFB4 expression but did not reach statistical significance (*p* = 0.1183).

**Table 1:** PFKFB4 expression and clinicopathological characteristics of patients with COAD.

Variables	Case, n	PFKFB4 expression		χ^2^	*p*-value
		Low, n (7)	High, n (72)		
Age, years					
≤ 62	40	25 (62.50)	15 (37.50)	1.9420	0.1634
> 62	39	30 (76.92)	9 (23.08)		
Gender					
Male	36	27 (75.00)	9 (25.00)	0.9050	0.3414
Female	43	28 (65.12)	15 (34.88)		
Type					
Others colon	45	36 (88.00)	9 (20.00)	5.3260	0.0210^a^
Sigmoid colon	34	19 (55.88)	15 (44.12)		
Differentiation					
Well + Moderate	40	36 (72.00)	14 (28.00)	0.2381	0.6256
Poor	27	18 (66.67)	9 (33.33)		
NA*	2	1 (50.00)	1 (50.00)		
Tumor size, cm					
≤ 4	45	32 (71.11)	13 (28.89)	0.1765	0.6744
> 4	33	22 (66.67)	11 (33.33)		
NA*	1	1 (100.00)	0 (0.00)		
pT stage					
T1 + 2	13	11 (84.62)	2 (15.38)	0.9750	0.3234
T3 + 4	65	43 (66.15)	22 (33.85)		
NA*	1	1 (100.00)	0 (0.00)		
pN stage					
N0	41	38 (74.51)	13 (25.49)	1.9270	0.1650
N1 + 2	27	16 (59.26)	11 (40.74)		
NA*	1	1 (100.00)	0 (0.00)		
pM stage					
M0	76	53 (69.74)	23 (30.26)		> 0.9999^b^
M1	3	2 (66.67)	1 (33.33)		
Dukes stage					
A + B	49	3 (75.51)	12 (24.49)	2.44	0.1183
C + D	29	17 (58.62)	12 (41.38)		
NA*	1	1 (100.00)	0 (0.00)		

^a^*P* < 0.05; ^b^Fisher’s exact test. NA, not applicable; *: means the degree of tumor differentiation, size, or stage of part of patients could not be defined. p, pathological; PFKFB4, phosphofructo‑2‑kinase/fructose‑2,6‑biphosphatase 4; COAD, colon adenocarcinoma.

### Higher PFKFB4 expression in COAD tissues than in adjacent normal tissues

**Figure 1:**
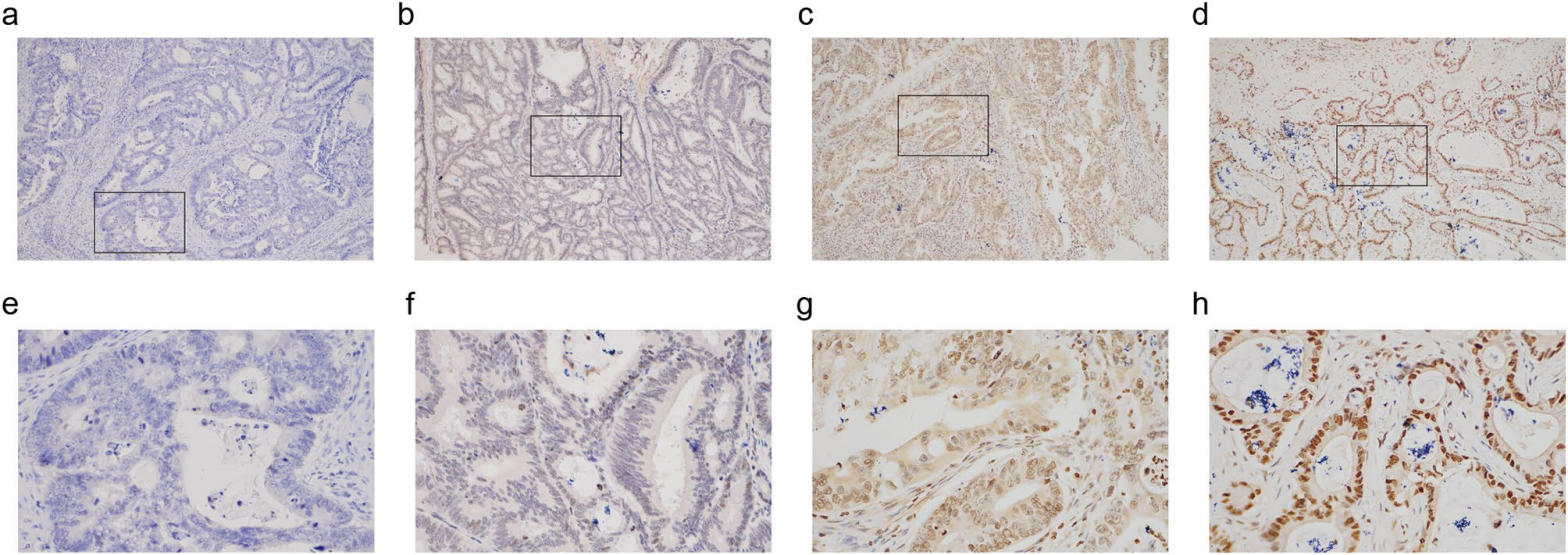
Representative immunohistochemical images of PFKFB4 expression in COAD tissues. This figure presents representative immunohistochemical images depicting the expression of PFKFB4 in COAD tissues. The images demonstrate the staining intensity at different magnifications: Negative staining (corresponding normal (control) tissue) at magnifications (**a**) × 100 and (**e**) × 400. Weak staining at magnifications (**b**) × 100 and (**f)** × 400. Moderate staining at magnifications (**c**) × 100 and (**g**) × 400. Strong positive staining at magnifications (**d**) × 100 and (**h**) × 400.

**Figure 2:**
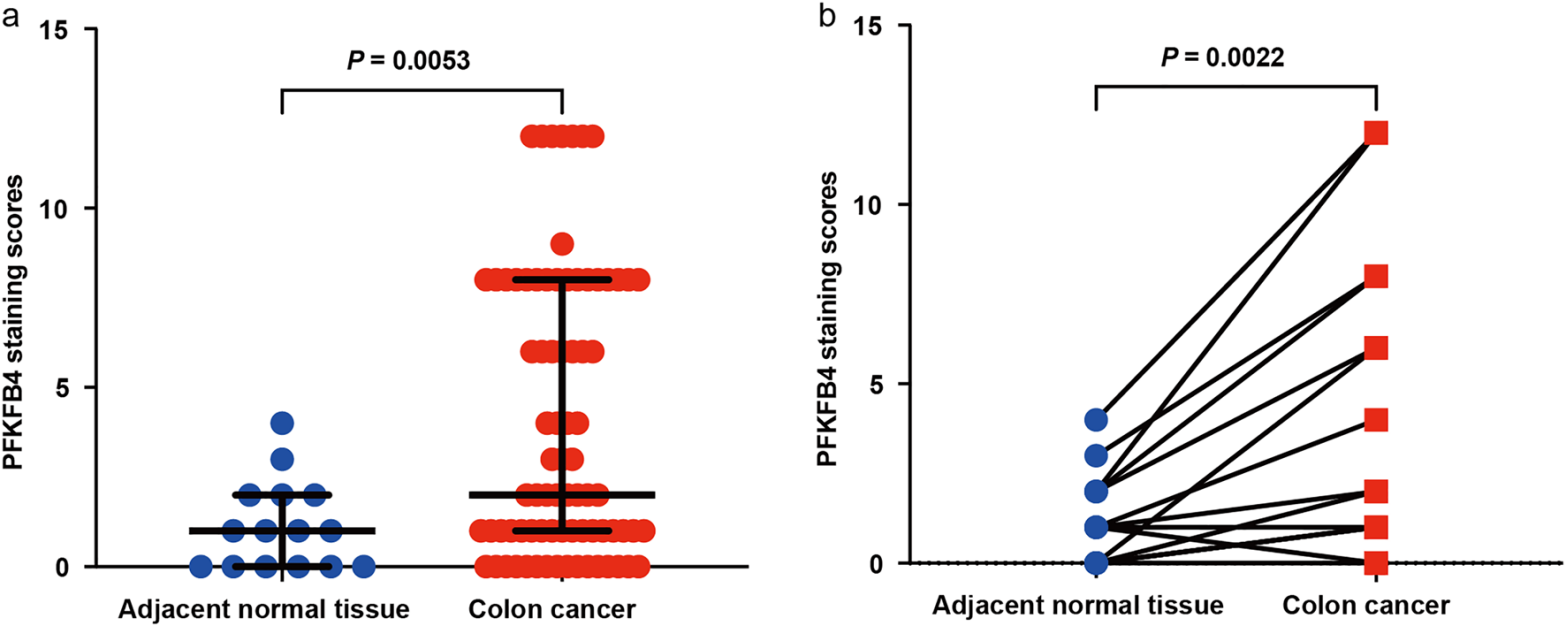
Elevated PFKFB4 expression in cancerous tissues compared with adjacent normal tissues. (**a**) The figure presents a box plot displaying the distribution of PFKFB4 staining scores in adjacent normal tissues (n = 15) and COAD tissues (n = 79). The box plot shows the median with the interquartile range. Statistical analysis was performed using the unpaired Student’s t-test to calculate the *p*-value. (**b**) A comparison of PFKFB4 expression between 15 pairs of matched neighboring normal tissues and COAD tissues. The analysis utilized the paired Student’s t-test to determine the *p*-value.

IHC analysis was performed to evaluate PFKFB4 expression levels in COAD tissues. Positive PFKFB4 expression was observed in nearly all tumor and adjacent normal tissues. Figure 1 shows representative IHC staining of PFKFB4 in colon cancer tissues. PFKFB4 was mainly expressed in the cytoplasm and nucleus, and it was diffusely distributed in the tumor tissues. The expression range and intensity of PFKFB4 were stronger in the nucleus than in the cytoplasm. Semi-quantification of PFKFB4 expression in COAD and adjacent normal tissues revealed significantly higher expression in tumor tissues compared to adjacent normal tissues (Fig. 2a). Moreover, PFKFB4 expression was significantly higher in COAD tissues than in matched adjacent normal tissues in the 15 pairs of matched samples analyzed (Fig. 2b).

### Differential expression analysis of PFKFB4 in COAD patients

**Figure 3:**
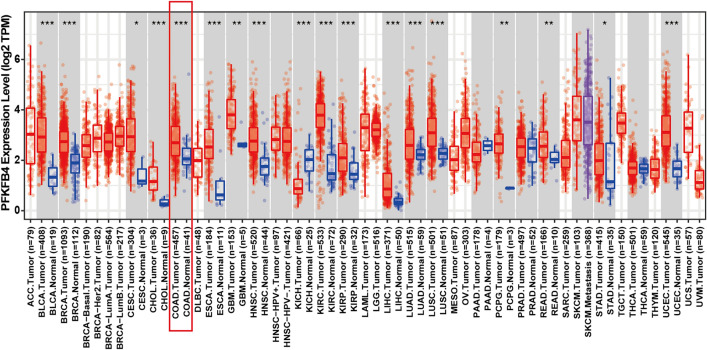
PFKFB4 expression in various cancer types. This figure depicts the expression levels of PFKFB4 in different types of cancer. The data for PFKFB4 expression were analyzed using the TIMER2.0 database, encompassing multiple cancer types. The significance levels for the analysis are represented as follows: *p < 0.05, **p < 0.01, and ***p < 0.001.

A pan-cancer analysis of PFKFB4 expression using the TIMER2.0 database demonstrated increased PFKFB4 mRNA levels in multiple malignancies, including COAD (Fig. 3).

### Prognostic analysis of PFKFB4 in COAD patients

**Figure 4:**
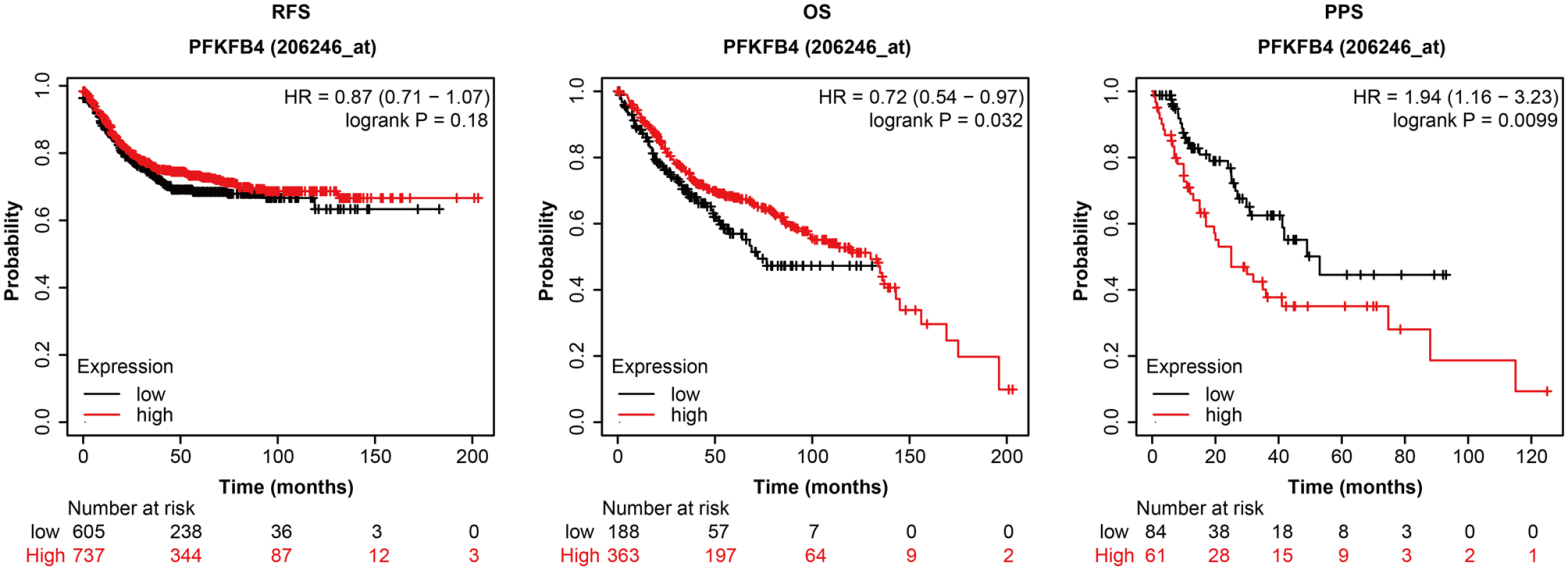
Relationship between high expression of PFKFB4 and RFS, OS, and PPS in COAD patients. This figure illustrates the association between high expression of PFKFB4 and three clinical outcomes in COAD patients: Relapse-Free Survival (RFS), Overall Survival (OS), and Post-Progression Survival (PPS). The analysis utilized Kaplan–Meier survival analysis on data obtained from the Kaplan–Meier plotter database. A subsequent log-rank test was performed to assess the statistical significance of the results.

By utilizing the online Kaplan–Meier plotter database, we investigated whether there existed a difference in survival time between COAD patients with high and low PFKFB4 expression. This comprehensive analysis aimed to provide deeper insights into the clinical significance of PFKFB4 in COAD patients. As shown in Fig. 4, the group with low PFKFB4 expression exhibited median survival times of 32 months for RFS, 72 months for OS, and 53 months for PPS. In contrast, the group with high PFKFB4 expression demonstrated median survival times of 42 months for RFS, 130 months for OS, and 25 months for PPS.

### Functional analysis of co-expressed genes of PFKFB4 in COAD patients

**Figure 5:**
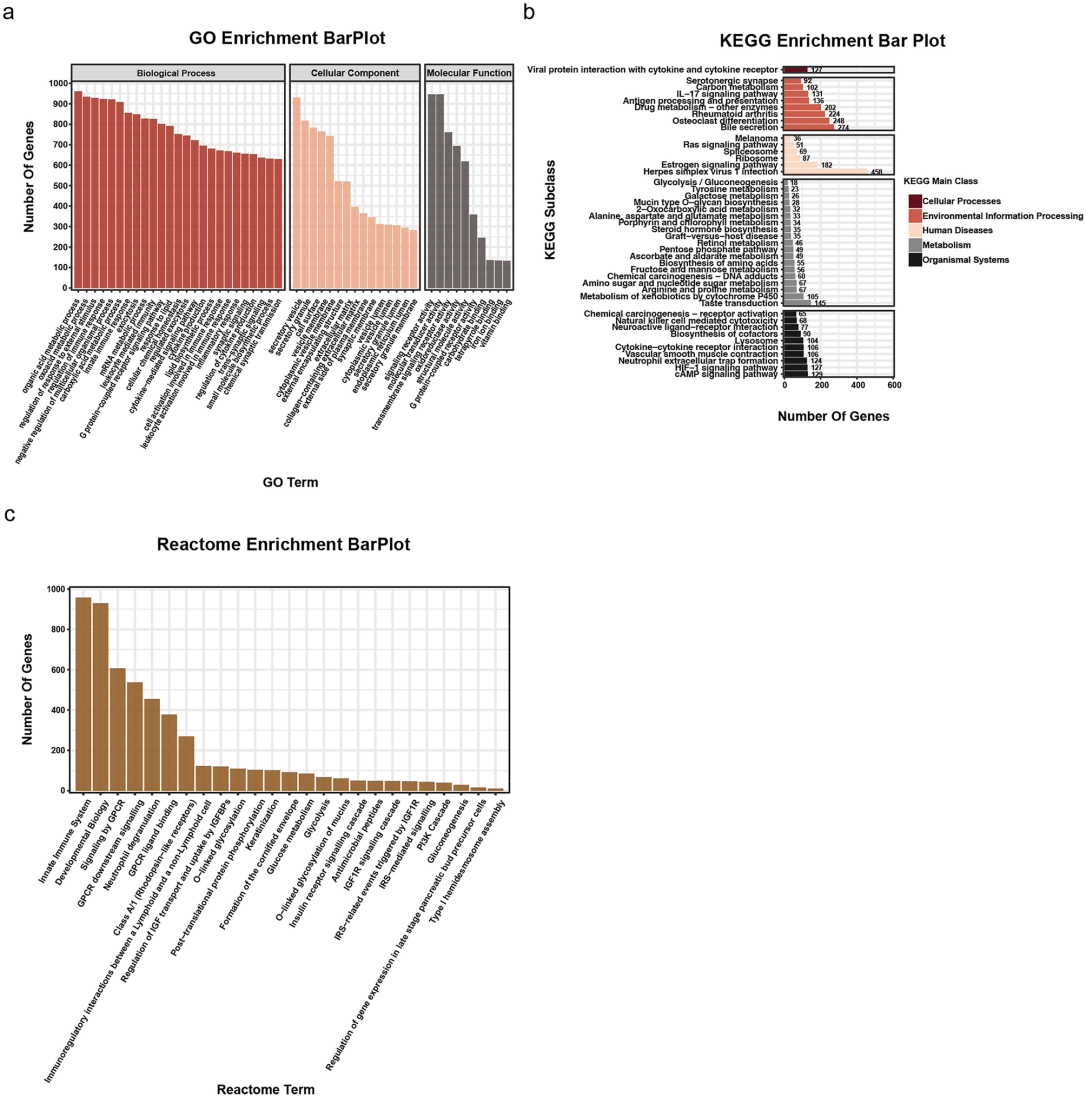
Histogram of functional analysis of co-expressed genes of PFKFB4 in COAD. In this figure, we present the functional analysis of co-expressed genes associated with PFKFB4 in COAD. A total of 10,189 PFKFB4-related genes in COAD were identified through co-expression analysis. These co-expressed genes were further subjected to classification and functional analyses using the following enrichment databases: (**a**) Gene Ontology (GO) enrichment analysis; (**b**) Kyoto Encyclopedia of Genes and Genomes (KEGG) enrichment analysis; (**c**) Reactome enrichment analysis. The CAMOIP database was used to perform these enrichment analyses.

**Figure 6:**
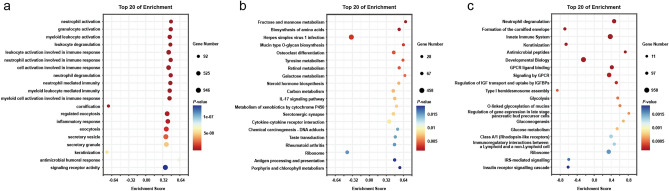
Bubble diagrams of functional analysis of co-expressed genes of PFKFB4 in COAD. This figure presents the functional analysis of co-expressed genes associated with PFKFB4 in COAD. A total of 10,189 PFKFB4-related genes in COAD were identified through co-expression analysis. The top 20 enriched pathways from the co-expressed genes of PFKFB4 were analyzed using the following enrichment databases: (**a**) Gene Ontology (GO) enrichment analysis; (**b**) Kyoto Encyclopedia of Genes and Genomes (KEGG) enrichment analysis; (**c**) Reactome enrichment analysis. The enrichment analyses were performed using the CAMOIP database.

Co-expression analysis using the CAMOIP database identified 10,189 PFKFB4-related genes in COAD. GO enrichment analysis of these genes revealed 271 associated functional annotations, comprising 190 for biological processes, 52 for cellular components, and 29 for molecular functions (Fig. 5a). KEGG pathway enrichment analysis showed 44 biological pathways, including 1 related to cellular processes, 8 to processing environmental information, 6 to human diseases, 19 to metabolism, and 10 to organic systems (Fig. 5b). Additionally, 26 Reactome functional annotations were obtained (Fig. 5c). The top 25 biological processes (BP), the top 15 cellular components (CC), and the top 10 molecular functions (MF) functional annotations were listed based on the Count values of the three classification functional annotations in GO, respectively. Enrichment analysis and display of these functional annotations were performed. The top 20 metabolic pathways with a count value in KEGG and the top 20 functional annotations with count value in Reactome were listed for enrichment analysis, and the bar chart was drawn. Correspondingly, bubble charts were created to show the top 20 functional annotations or pathways from GO, KEGG, and Reactome, respectively (Fig. 6).

The top five GO functional annotations related to PFKFB4 in COAD are *neutrophil activation*, *granulocyte activation*, *myeloid leukocyte activation*, *leukocyte degranulation*, and leukocyte activation involved in immune response. The top five pathways on the PFKFB4-related KEGG pathways list are *fructose and mannose metabolism*, *biosynthesis of amino acids*, *herpes simplex virus 1 infection*, *mucin type O-glycan biosynthesis*, and *osteoclast differentiation*. The top five Reactome functional annotations associated with PFKFB4 are *neutrophil degranulation*, *formation of the cornified envelope*, *innate immune system*, *keratinization*, and *antimicrobial peptides*.

### The PFKFB4 expression and immune infiltration level in COAD patients

Using the TIMER2.0 database, we investigated whether PFKFB4 expression contributes to the development of COAD by affecting immune cell infiltration. Our analysis showed that PFKFB4 gene expression in CRC was significantly correlated with the infiltration of CD8+ T cells, CD4+ T cells, regulatory T cells (Tregs), macrophages, neutrophils, dendritic cells, and natural killer (NK) cells (Supplementary Table S1).

**Figure 7:**
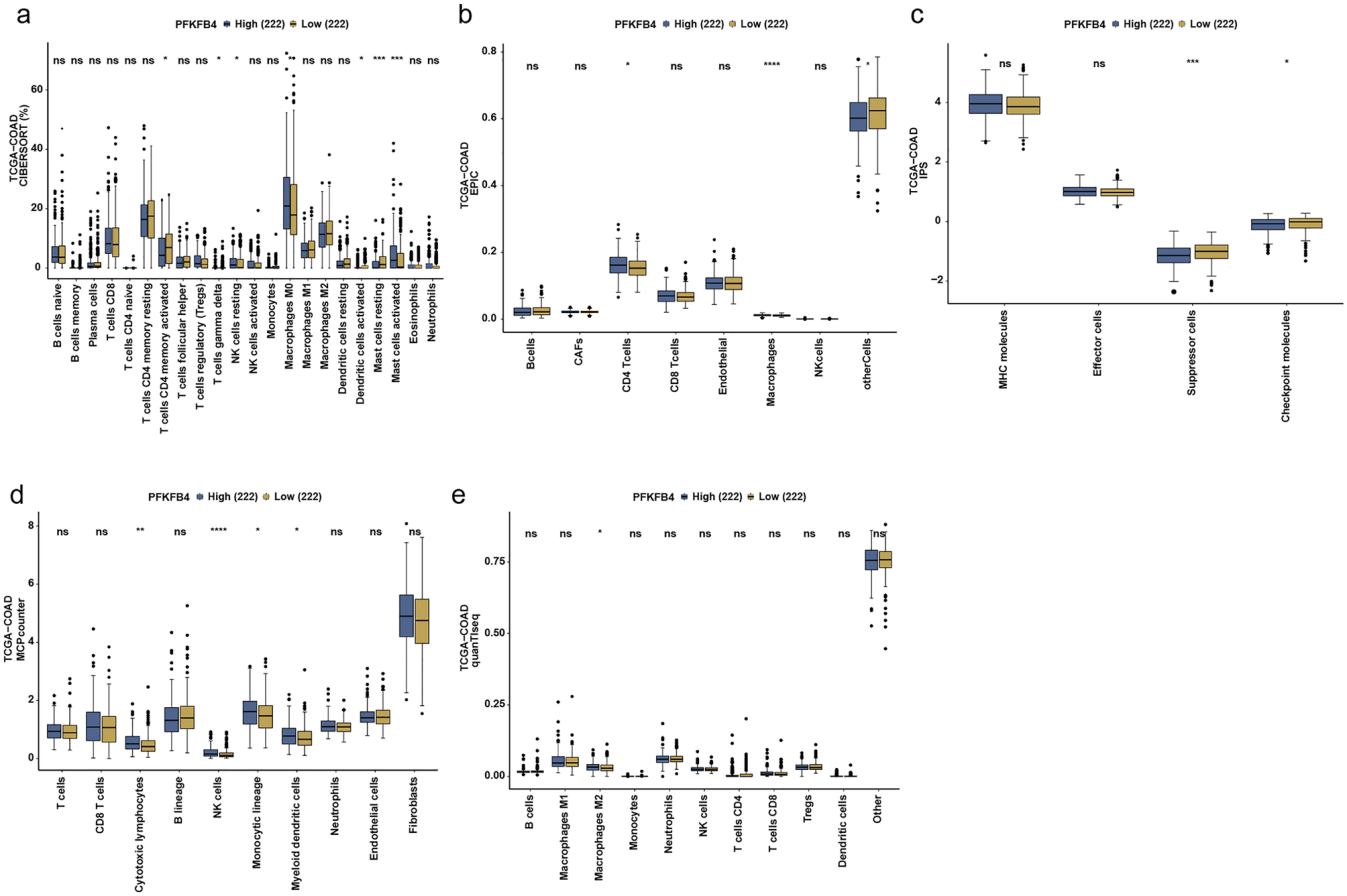
Correlation of PFKFB4 expression with immune infiltrates in COAD. This figure demonstrates the correlation between PFKFB4 expression and immune infiltrates in COAD. The immune cell scores estimated by different methods were compared between different groups using the Mann–Whitney U-test based on the CAMOIP database. Comparison of immune cell scores estimated by (**a**) CIBERSORT, (**b**) EPIC, (**c**) IPS, (**d**) MCPcounter, and (**e**) quanTIseq, respectively. The significance levels for the comparisons are indicated as follows: *p < 0.05, **p < 0.01, ***p < 0.001, and ****p < 0.0001.

Furthermore, we analyzed TCGA-COAD data using the CAMOIP database to explore the impact of PFKFB4 gene levels on various immune cells. Our results indicated a positive correlation between PFKFB4 expression and the infiltration of CD4+ T cells, gamma delta T cells, macrophages, myeloid dendritic cells, activated mast cells, and resting NK cells in COAD (Fig. 7).

## Discussion

Most cancer cells generate energy through a process known as “aerobic glycolysis,” characterized by high glucose uptake and glycolysis, followed by lactic acid fermentation in the cytosol, rather than the oxidative phosphorylation and citric acid cycle observed in normal cells^13^. Numerous human cancers, including glioblastoma, gastric cancer, pancreatic cancer, bladder cancer, and SCNC, exhibit elevated expression levels of PFKFB4, one of the four isoenzymes of phosphofructokinase^14^. PFKFB4 regulates glycolysis in cells by generating the glycolytic signaling molecule F-2,6-BP and subsequently hydrolyzing it, thus limiting tumor development^15^. However, an increasing number of studies have uncovered a connection between the occurrence, growth, and prognosis of specific malignancies and the overexpression of PFKFB4 in different tumors^16^. For instance, patients with low PFKFB4 expression in gastric cancer had significantly longer overall survival (OS), initial progression survival, and post-progression survival durations compared to those with high PFKFB4 expression^17^.

PFKFB4 plays a pivotal role in controlling the metabolic fluxes of the glycolytic and pentose phosphate pathways (PPP), which are the primary mechanisms tumor cells utilize to metabolize glucose. Studies have shown that PFKFB4 controls the formation of reactive oxygen species (ROS) by directing glucose metabolic intermediates to the PPP in various cancer cells^16,18,19^. Moreover, PFKFB4 by reducing prostate cancer cells fructose-2,6-bisphosphate level, make the glucose-6-phosphate guide the PPP, reduces the glycolytic pathway activity, make the NADPH and glutathione levels, eventually reduce the oxidative stress and death of tumor cells, prevent prostate cancer cells apoptosis, promote the growth of tumor^20^.

In addition to its role in glycolysis, glycolytic enzymes are now recognized to participate in various physiological and pathological processes^21^. Notably, PFKFB4 expression was significantly higher in solid breast cancers compared to healthy tissues. Researchers have found that the protein kinase PFKFB4 phosphorylates the oncogenic steroid receptor coactivator-3 (SRC-3), enhancing its transcriptional activity and promoting the development of breast cancer^22^. Furthermore, PFKFB4 is involved in regulating the cell cycle, autophagy, and tumor metastasis^23^. Nevertheless, few studies have investigated the relationship between PFKFB4 and COAD.

This study has identified a potential correlation between COAD and PFKFB4 expression in clinical samples. The results indicate that COAD tissues exhibit significantly higher levels of PFKFB4 expression compared to adjacent normal tissues. Furthermore, patients with COAD in the sigmoid colon had considerably greater levels of PFKFB4 expression than those with COAD in other parts of the colon. This may be linked to the fact that sigmoid colon is the most common site of COAD, which is located in the right colon and tends to be more malignant^24^. However, the investigation did not find any direct relationship between PFKFB4 expression and patient age, gender, tumor differentiation, size, depth of invasion, lymph node metastasis, or distant metastasis. This could be due to the inadequate samples.

The clinical samples collected in this study demonstrated a high expression of PFKFB4 in COAD patients. Regarding the prognosis of COAD patients, the corresponding data revealed that high expression of PFKFB4 was associated with improved OS. This finding suggests that COAD patients with high PFKFB4 expression may have better survival rates after treatment, indicating a positive correlation with prognosis before disease progression.

However, intriguingly, the data also showed that PPS was worse in COAD patients with high PFKFB4 expression. This suggests that after disease progression in COAD patients, high expression of PFKFB4 may be linked to shorter survival and an unfavorable prognosis. This finding implies that high expression of PFKFB4 might play a role in promoting the late progression of COAD.

To delve deeper into this seemingly contradictory result, we first conducted gene enrichment analysis of the co-expressed genes associated with PFKFB4 using the CAMOIP database, followed by functional annotation and enrichment analysis. This analysis revealed several PFKFB4-related pathways in COAD, including up-regulation of *granulocyte activation*, *myeloid leukocyte activation*, *inflammatory response*, *biosynthesis of amino acids*, *fructose and mannose metabolism*, *glycolysis*, *gluconeogenesis*, and *glucose metabolism*. These findings suggest that COAD patients with high PFKFB4 expression may have a higher tendency to utilize the glycolytic pathway for metabolic biosynthesis, which could be associated with enhanced immunological responses and increased immune infiltration.

Proverbially, the tumor microenvironment (TME) influences the development and recurrence of tumors, and immune cells in TME have been found to either promote or prevent tumor growth^25,26^. Moreover, immune infiltration, a key element of the TME, has been shown to impact tumor development and response to therapy^27^. Using the TIMER2.0 and CAMOIP databases, we observed a robust correlation between PFKFB4 expression and the levels of infiltration of various immune cells, including CD8+ T cells, CD4+ T cells, regulatory T cells (Tregs), macrophages, neutrophils, dendritic cells, activated mast cells, and resting NK cells. This suggests that PFKFB4 may also be a marker for immune status and provide information for immunotherapies.

Accordingly, we speculate that the relationship between high expression of PFKFB4 and prognosis of COAD patients is complex and diverse, and it may play different roles in different stages of COAD, leading to the seemingly contradictory results between OS and PPS. This seemingly paradoxical result may be due to the complex biological function of PFKFB4 in COAD. High expression of PFKFB4 may be beneficial to inhibit the proliferation of tumor cells in the initial stage, thereby associated with better OS. However, when the disease progresses to an advanced stage, PFKFB4 may begin to play a role in promoting tumor progression, resulting in a lower prognostic value for post-progression survival. These results may involve a variety of metabolic pathways such as *biosynthesis of amino acids*, *glycolysis*, *gluconeogenesis*, *glucose metabolism*, and *inflammatory response*, and a variety of infiltration of immune cells in the body, such as CD8+ T cells, CD4+ T cells, Tregs, macrophages, neutrophils, dendritic cells, active mast cells, and resting NK cells. They work together to produce a complex diversity of the relationship between PFKFB4 expression and the prognosis of COAD patients.

5-FU is a chemotherapeutic agent used in the treatment of colon cancer. However, it can also indirectly affect glycolysis by disrupting the DNA repair process, leading to cell death. In addition, several studies have shown that alterations in glycolytic metabolism may affect the sensitivity of cancer cells to chemotherapeutic agents such as 5-FU. In some cases, increased glycolysis was associated with increased 5-FU sensitivity^28^. The effect of 5-FU on DNA can indirectly affect the glycolysis pathway in cancer cells. PFKFB4, as a glycolytic regulator, may play a role in determining the response of tumor cells to 5-FU treatment. The balance between glycolysis and response to chemotherapy is an ongoing area of research in oncology^29^. Clearly, this study may provide relevant ideas and evidence for the role of PFKFB4 in the anti-colon cancer mechanism of 5-FU.

Unfortunately, our study has some limitations. Firstly, our clinical data might be affected by sampling errors due to the limited sample size. Secondly, although we used the TCGA database for additional analysis to address the issue of inadequate sample size, the amount of PFKFB4 gene samples in COAD remains insufficient. Furthermore, based on informatics analysis for clinical databases, it is reasonable to draw this conclusion. Admittedly, this is also the deficiency of our study, and we will conduct experimental analysis at the cellular level in the follow-up research plan in order to further verify our conclusion. Therefore, more research is needed to validate our findings and explore further the molecular linkages, clinical applications, and mechanisms of PFKFB4 in COAD.

## Conclusion

The expression of PFKFB4 is significantly higher in COAD tissue, with particularly elevated levels observed in the sigmoid colon compared to other regions of the colon. Moreover, the relationship between high PFKFB4 expression and the prognosis of COAD patients is intricate and diverse, as it may assume distinct roles at different stages of COAD. The underlying mechanism may involve multiple metabolic pathways, including *biosynthesis of amino acids*, *glycolysis*, *gluconeogenesis*, *glucose metabolism*, and *inflammatory response*. Future studies are essential to gain a comprehensive understanding of the function and potential therapeutic significance of PFKFB4 in COAD. These investigations will provide valuable insights and contribute to a better prognostic evaluation of COAD patients.

## Materials and methods

### Patients and specimens

Tumor tissue samples were collected from 79 patients diagnosed with colon COAD who were admitted to Ningxia Medical University General Hospital (Yinchuan, China) between September 2016 and December 2017. Among these patients, only 15 tumor tissues had matched adjacent normal tissues (located at least 2 cm from the tumor margin) due to the tissue section generation process. The samples were fixed with 4% polyformaldehyde at room temperature for 24 h and subsequently embedded in paraffin wax. The tissues were then sectioned into 5-µm-thick slices for immunohistochemical (IHC) analysis. Pathological diagnosis was performed by two senior pathologists. Clinicopathological data of the patients are presented in Table 1, although 2 cases lacked information on tumor differentiation, and 1 case lacked information on tumor size, depth of invasion, lymph node metastasis, and Dukes’ stage. The experiments involving human subjects were carefully examined and authorized by the Medical Research Ethics Review Committee of the General Hospital of Ningxia Medical University (Yinchuan, China). The participants/patients provided written informed consent prior to their participation in the investigation. All research was carried out in accordance with relevant guidelines and regulations.

### Immunohistochemical staining

The expression levels of PFKFB4 in COAD tissues were determined using immunohistochemical (IHC) analysis. Formalin-fixed and paraffin-embedded tissue sections were heated at 65 °C for 1 h, followed by deparaffinization in xylene and rehydration in a series of ethanol solutions. To block endogenous peroxidase activity, the sections were treated with 3% hydrogen peroxide for 10 min at room temperature after three washes with PBS. Antigen retrieval was performed using EDTA buffer (pH 8.0). Subsequently, the sections were blocked with 10% goat serum (Cat. No. ZLI-9022; OriGene Technologies) for 10 min at 37 °C. Next, the sections were incubated with anti-PFKFB4 antibody (1:100; Cat. No. ab137785; Abcam) at 37 °C for 1 h, followed by overnight incubation in a humid environment at 4 °C. The next day, the sections were washed three times with PBS and then incubated with HRP-conjugated peroxidase working solution (Cat. No. P0448; Dako; Agilent Technologies, Inc.) for 30 min at 37 °C. After treating the sections with the chromogen substrate 3,3′-diaminobenzidine for 10 min at room temperature, counterstaining with hematoxylin was performed for 1 min at room temperature. The slides were then dehydrated in progressively higher concentrations of alcohol and xylene before being sealed with neutral balsam and glass coverslips.

To assess PFKFB4 expression levels, a semi-quantitative method was used, where a light microscope was employed to view and capture images of the sections^17,30^. Two experienced pathologists graded the intensity of staining as follows: 0 for negative, 1 for light yellow, 2 for tan, and 3 for brown. Additionally, the total number of stained cells was scored based on the proportion of positively stained cells, which were categorized into four groups: 0 for no positively stained cells, 1+ for ≤ 10% positive cells, 2+ for 11–50% positive cells, 3+ for 51–75% positive cells, and 4+ for > 75% positively stained cells. The final score was determined by multiplying the staining intensity score and the proportion of positively stained cells score. To distinguish samples with high and low expression, a cutoff value based on the median IHC score was used.

### Expression analysis

To address statistical bias caused by the limited sample size in the analysis of PFKFB4 expression in COAD, we employed the TIMER2.0 online platform (http://timer.cistrome.org/) to access the PFKFB4 expression data across various tumors and evaluate the expression in adjacent non-cancerous tissues^31^.

### Survival analysis

We conducted survival analysis for COAD using the Kaplan–Meier plotter database (https://kmplot.com/analysis) and performed a log-rank test^32^. The survival analysis focused on Affymetrix ID 206246_at, which corresponds to PFKFB4. The assessment included a total of 1302 COAD patients for relapse-free survival (RFS), 550 patients for overall survival (OS), and 145 patients for post-progression survival (PPS).

### Functional enrichment analysis

We utilized the CAMOIP online platform to identify genes associated with PFKFB4 in COAD based on Cancer Genome Atlas (TCGA)-COAD data [16]. Our screening criteria for these genes were set at *p*-values < 0.05 and FDR (q-value) < 0.25. Detailed annotation information about Kyoto Encyclopedia of Genes and Genomes (KEGG), Reactome, and Gene Ontology biological process (GO) was obtained for these selected genes. Visualization of the associated functional annotations or pathways was achieved using the resources provided by the web platform, in the form of a histogram (available at https://www.omicsstudio.cn) and a bubble chart (available at https://cloud.oebiotech.cn), respectively.

### Analysis of immune cell infiltration

To investigate the association between PFKFB4 expression and immune cell infiltration in COAD, we utilized the TIMER2.0 database and the CIBERSORT algorithm^31^. Tumor purity-adjusted partial Spearman’s correlation was used to evaluate the relationship between PFKFB4 mRNA expression levels and immune infiltration levels. Statistical significance was considered at a *p*-value threshold of < 0.05.

Additionally, we employed the CAMOIP database and the CIBERSORT algorithm to assess the immune cells associated with PFKFB4 expression in COAD^33^. The CIBERSORT algorithm allowed us to evaluate the following immune cell types: B cells naive, B cells memory, plasma cells, T cells CD8+, T cells CD4+ naive, T cells CD4+ memory resting, T cells CD4 memory activated, T cells follicular helper, T cells regulatory (Tregs), T cells gamma delta, NK cells resting, Monocytes, Macrophages M0, M1, and M2, Mast cells resting, Mast cells active, Dendritic cells resting, Dendritic resting cells, Dendritic activated cells, Neutrophils, and Eosinophils^34^. Moreover, we used the EPIC algorithm to score B cells, cancer-associated fibroblasts (CAFs), CD4+ T cells, CD8+ T cells, endothelial cells, macrophages, NK cells, and other cells. The IPS algorithm allowed us to rate MHC molecules, effector cells, suppressor cells, and checkpoint molecules^35^. Finally, we employed the MCPcounter method to evaluate T cells, CD8+ T cells, cytotoxic lymphocytes, B lineage, NK cells, monocytic lineage, myeloid dendritic cells, neutrophils, endothelial cells, and fibroblasts^36^. Additionally, we used the quanTIseq method to appraise B cells, macrophages M1 and M2, monocytes, neutrophils, NK cells, T cells CD4+ and CD8+ , Tregs, dendritic cells, and other immune cells^37^.

### Statistical analysis

Statistical analysis and graphical representations were performed using GraphPad Prism 9.4 (La Jolla, CA, USA). The relationship between PFKFB4 expression and clinicopathological features was assessed using either a χ^2^ test or Fisher’s exact test. We employed a two-tailed Student’s t-test to compare COAD tissues and adjacent normal tissues, while the Mann–Whitney U test was used to compare immune cells between the PFKFB4 high expression group and low expression group. Statistical significance was defined as *p* < 0.05.

### Supplementary Information

Supplementary Table S1.

## Data Availability

The authors of this study will provide the clinical data sets used or analyzed in the investigation upon a reasonable request. The study utilized publicly accessible datasets, which can be found on the websites of TIMER2.0 (http://timer.cistrome.org/), Kaplan–Meier plotter (https://kmplot.com/analysis), and CAMOIP (http://www.camoip.net/). All necessary information can be accessed through these websites.
