# Author Correction: Differential roles of highly expressed PFKFB4 in colon adenocarcinoma patients

**DOI:** 10.1038/s41598-023-49212-z

**Published:** 2023-12-09

**Authors:** Xiaojing Gu, Xingchen Dai, Yongli Huang, Yuhuan Zhang, Lintao Dong, Chanchan Gao, Fang Wang

**Affiliations:** 1 Department of Gastroenterology, General Hospital, Ningxia Medical University, Yinchuan, Ningxia China; 2 School of Clinical Medicine, Ningxia Medical University, Yinchuan, Ningxia China; 3 Key Laboratory of Fertility Preservation and Maintenance of Ministry of Education, Department of Biochemistry and Molecular Biology, School of Basic Medical Sciences, Ningxia Medical University, Yinchuan, Ningxia China; 4 Department of Oncology, Zhongda Hospital, Southeast University, Nanjing, Jiangsu China

Correction to: *Scientific reports* 10.1038/s41598-023-43619-4, published online 28 September 2023

The original version of this Article contained errors.

In the Abstract,

“Interestingly, high PFKFB4 expression was associated with both improved overall survival (OS) and worse progression-free survival (PPS) in COAD patients.”

now reads:

“Interestingly, high PFKFB4 expression was associated with both improved overall survival (OS) and post-progression survival (PPS) in COAD patients.”

In addition, Figure 4 depicted the correlation of PFKFB4 expression with patient outcomes in different stages (Stage II, III, and IV based on TNM staging), instead of the associations between PFKFB4 expression levels and various survival outcomes (Relapse-Free Survival [RFS], Overall Survival [OS], and Post-Progression Survival [PPS]) in patients with Colon Adenocarcinoma (COAD).

The original Figure 4 and accompanying legend appear below.

**Figure 4:**
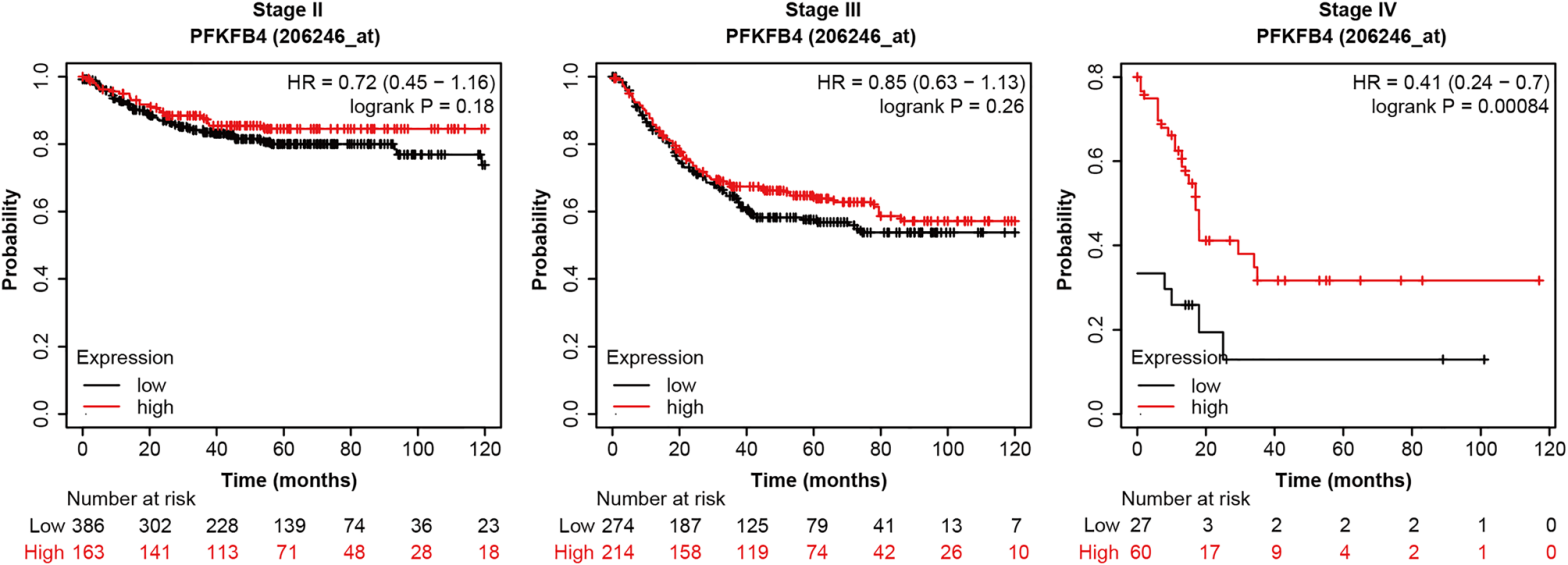
Relationship between high expression of PFKFB4 and RFS, OS, and PPS in COAD patients. This figure illustrates the association between high expression of PFKFB4 and three clinical outcomes in COAD patients: Relapse-Free Survival (RFS), Overall Survival (OS), and Post-Progression Survival (PPS). The analysis utilized Kaplan–Meier survival analysis on data obtained from the Kaplan–Meier plotter database. A subsequent log-rank test was performed to assess the statistical significance of the results.

The original Article has been corrected.

