# Supplementary material for: Differential roles of highly expressed PFKFB4 in colon adenocarcinoma patients
Source: Sci Rep. 2023 Sep 28;13:16284. doi: 10.1038/s41598-023-43619-4 (PMC10539362; doi:10.1038/s41598-023-43619-4)
Supplement: Supplementary file 1 — Supplementary Table S1. [file 41598_2023_43619_MOESM1_ESM.docx]

**Table S1. Correlation between PFKFB4 expression and infiltration level of immune cells in COAD using TIMER2.0 database.**

| Infiltrates | Rho value* | *p*-value** |
| --- | --- | --- |
| Myeloid dendritic cell (DC) | 0.344558 | 4.40E-09 |
| Natural Killer (NK) cell | 0.312652 | 1.19E-07 |
| Neutrophil | 0.295711 | 5.93E-07 |
| M2 Macrophage | -0.24125 | 8.71E-07 |
| CD8+ T cell | 0.284013 | 1.69E-06 |
| Macrophage | 0.259129 | 1.35E-05 |
| M1 Macrophage | 0.255989 | 1.73E-05 |
| Monocyte | 0.22559 | 0.000161 |
| resting Mast cell | 0.224431 | 0.000175 |
| Regulatory T cell (Tregs) | 0.221872 | 0.000208 |
| M0 Macrophage | 0.216674 | 0.000295 |
| Macrophage/Monocyte | 0.214593 | 0.000338 |
| Cancer associated fibroblast | 0.199824 | 0.000861 |
| plasma B cell | -0.19572 | 0.001105 |
| Common lymphoid progenitor | -0.19366 | 0.001249 |
| memory resting CD4+ T cell | 0.190606 | 0.001495 |
| effector memory CD4+ T cell | 0.186765 | 0.001868 |
| Th2 CD4+ T cell | 0.182962 | 0.002319 |
| Hematopoietic stem cell | -0.18234 | 0.002401 |
| naive CD8+ T cell | -0.17632 | 0.003351 |
| CD4+ T cell | 0.155609 | 0.009752 |
| Granulocyte-monocyte progenitor | 0.152455 | 0.011358 |
| gamma delta T cell | -0.14725 | 0.014519 |
| Endothelial cell | 0.143062 | 0.017604 |
| MDSC | -0.11428 | 0.021268 |
| central memory CD8+ T cell | 0.134576 | 0.025635 |
| memory CD4+ T cell | 0.124805 | 0.038609 |
| Common myeloid progenitor | -0.11441 | 0.058106 |
| activated Natural Killer (NK) cell | 0.114231 | 0.058508 |
| memory B cell | -0.11333 | 0.060531 |
| Class-switched memory B cell | 0.105464 | 0.080841 |
| activated Myeloid dendritic cell (DC) | -0.10079 | 0.095316 |
| naive B cell | -0.08665 | 0.151858 |
| follicular helper T cell | 0.081435 | 0.178132 |
| B cell | 0.063949 | 0.290639 |
| NK T cell | 0.05927 | 0.327453 |
| memory activated CD4+ T cell | 0.054442 | 0.368456 |
| non-regulatory CD4+ T cell | 0.054289 | 0.369799 |
| Th1 CD4+ T cell | 0.043799 | 0.469454 |
| central memory CD4+ T cell | -0.04337 | 0.473825 |
| Eosinophil | -0.03714 | 0.539647 |
| Mast cell | 0.034141 | 0.572921 |
| naive CD4+ T cell | 0.031614 | 0.601665 |
| resting Natural Killer (NK) cell | 0.007353 | 0.903392 |
| activated Mast cell | 0.005561 | 0.926852 |
| resting Myeloid dendritic cell (DC) | 0.002293 | 0.969808 |
| effector memory CD8+ T cell | -0.00034 | 0.99549 |

* Rho value of Spearman’s correlation, rho value was adjusted by purity. ** *p*-value < 0.05 was considered statistically significant. PFKFB4: phosphofructo‑2‑kinase/fructose‑2,6‑biphosphatase 4; COAD: Colon Adenocarcinoma.
